# Author Correction: A soft, self-sensing tensile valve for perceptive soft robots

**DOI:** 10.1038/s41467-023-40295-w

**Published:** 2023-07-31

**Authors:** Jun Kyu Choe, Junsoo Kim, Hyeonseo Song, Joonbum Bae, Jiyun Kim

**Affiliations:** 1 Department of Materials Science and Engineering, Ulsan National Institute of Science and Technology (UNIST), Ulsan, 44919 Republic of Korea; 2 Department of Mechanical Engineering, Ulsan National Institute of Science and Technology (UNIST), Ulsan, 44919 Republic of Korea; 3 Center for Multidimensional Programmable Matter, Ulsan National Institute of Science and Technology, Ulsan, 44919 South Korea

**Keywords:** Polymers, Mechanical engineering

Correction to: *Nature Communications* 10.1038/s41467-023-39691-z, published online 04 July 2023

The original version of this Article contained an error in Fig. 2, in which Fig 2b’s arrow with the word “Extension” is unclear due to the arrow colour formatting and Fig 2h also has unclear “inversed” dataset due to the light colour format. The correct version of Fig. 2 is:
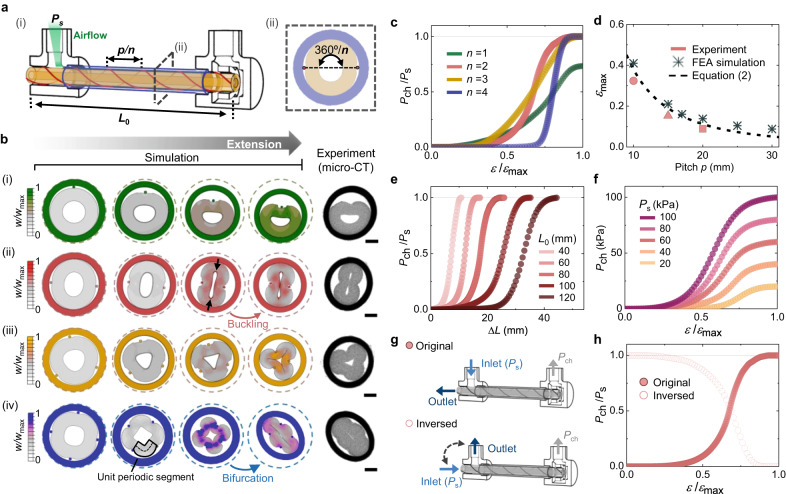


which replaces the previous incorrect version:


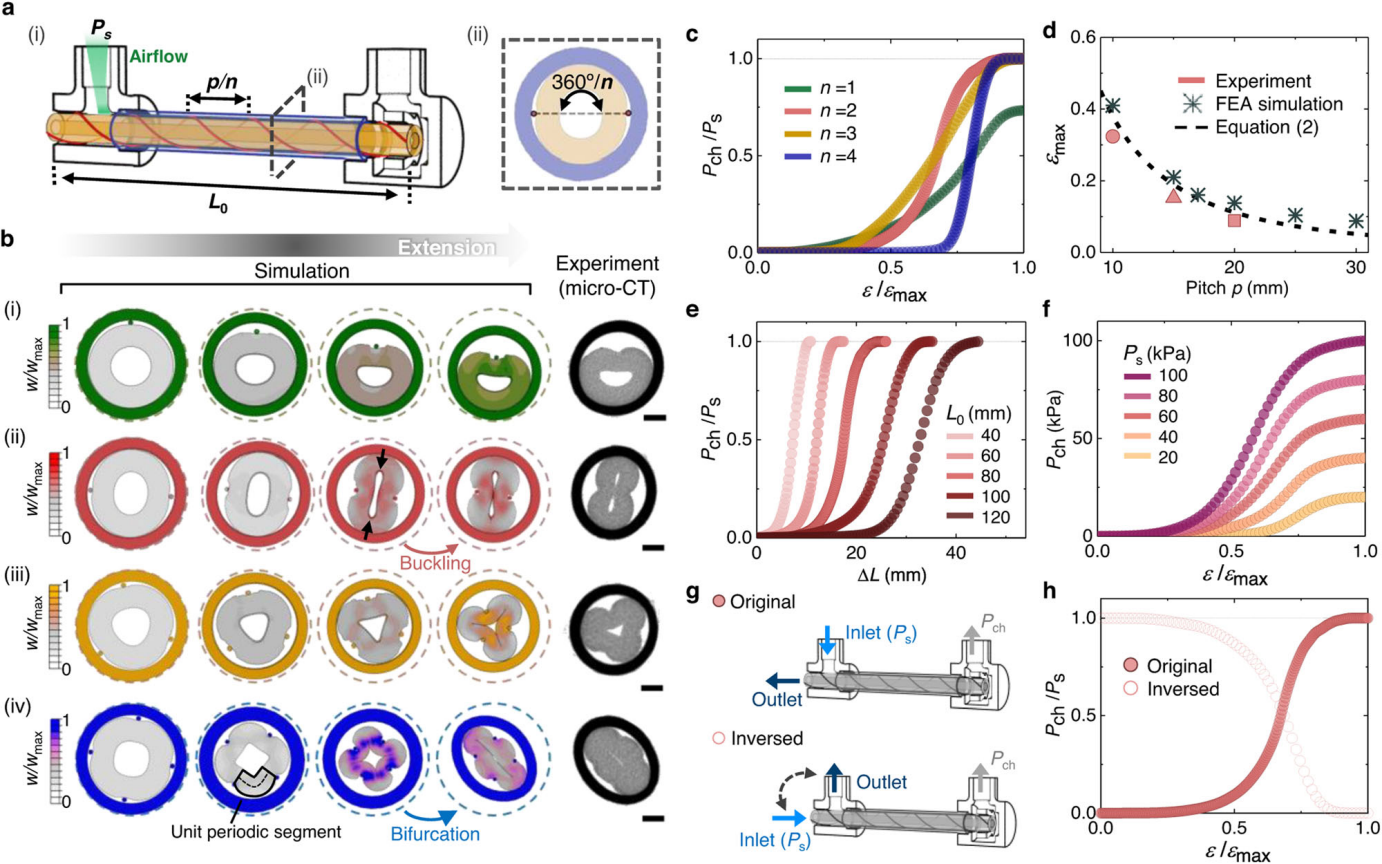


This has been corrected in both the PDF and HTML versions of the Article.

