# Supplementary material for: A soft, self-sensing tensile valve for perceptive soft robots
Source: Nat Commun. 2023 Jul 4;14:3942. doi: 10.1038/s41467-023-39691-z (PMC10319868; doi:10.1038/s41467-023-39691-z)
Supplement: Supplementary file 1 — Supplementary information [file 41467_2023_39691_MOESM1_ESM.pdf]

Supporting Information:

A Soft, Self-Sensing Tensile Valve for Perceptive Soft Robotics

Jun Kyu Choe[1,4], Junsoo Kim[2,4], Hyeonseo Song[1], Joonbum Bae[2] *, Jiyun Kim[1,3] *

1 Department of Materials Science and Engineering, Ulsan National Institute of Science and Technology (UNIST), Ulsan 44919, Republic of Korea.

2 Department of Mechanical Engineering, Ulsan National Institute of Science and Technology (UNIST), Ulsan 44919, Republic of Korea.

3 Center for Multidimensional Programmable Matter, Ulsan National Institute of Science and Technology, Ulsan 44919, South Korea

4 These authors contributed equally to this work: Jun Kyu Choe and Junsoo Kim

*Email: jbbae@unist.ac.kr (J.B.); jiyunkim@unist.ac.kr (J.K.)

In this supplementary document, we provide (Supplementary Note 1) the calculation of the large deformation equation to predict the deformation of the tubes, (Supplementary Note 2) the derivation of the maximum strain and maximum extension length equations, (Supplementary Note 3) the considerable factors of the STV during practical use, (Supplementary Fig. 1-13), (Supplementary Table. 1-4), and (Supplementary References).

Supplementary Note 1: Deformation contour

To predict the nonlinear deformation behavior of the inner tube under nonuniform radial pressure, the applied pressure is assumed to have the following form:

$$\bar{P} = \bar{P}_0\left(1 + \cos\left(n\tilde{\theta}\right)\right), \tag{S1}$$

where $\bar{P}_0$ is the average pressure parameter over the inner tube centerline circumference, n is the number of WHYs wrapped onto the inner tube with equal radial distributions, and $\tilde{\theta}$ is the angle between the normal to the centerline of the undeformed inner tube and the horizontal axis.

The centerline of the inner tube is assumed to be inextensible with a change in curvature proportional to the applied bending moment. Additionally, it is assumed that there is no axial or shear deformation of the inner tube but only bending deformation of the inner tube. The governing equation for this problem has been shown using large deformation thin beam theory[1] as follows:

$$\frac{d^2U}{d\tilde{\theta}^2} + \frac{1}{2}U^3 + CU = \bar{P}, \tag{S2}$$

where $U = R/\rho$ is the ratio between the radius of curvature before deformation R and the radius of curvature after deformation ρ, and C is a constant $C = (\bar{P}_0 - \frac{1}{4\pi}\int_0^{2\pi} U^3 d\tilde{\theta})$. Assuming inextensibility where the undeformed length of the tube segment $\mathrm{d}s = R\mathrm{d}\tilde{\theta}$ is the same as the deformed length of the tube segment $\mathrm{d}s = \rho\mathrm{d}\tilde{\theta}$, we obtain the relationship $U = R\mathrm{d}\phi/\mathrm{d}s = \mathrm{d}\phi/\mathrm{d}\tilde{\theta}$.

Since equation (S2) is a highly nonlinear Duffing equation, a perturbation solution is attained by assuming the following Taylor series expansions:

$$U = 1 + \eta v_1 + \eta^2 v_2 + \eta^3 v_3 + \cdots, \tag{S3}$$

$$\bar{P}_0 = \eta A_1 + \eta^2 A_2 + \eta^3 A_3 + \cdots, \tag{S4}$$

where η is the perturbation variable, A_i are constants, and v_i are functions of $\tilde{\theta}$ which are periodic. Inserting equation (S3) and equation (S4) into equation (S2) and equating different powers of η with U and $\bar{P}_0$ calculated up to second and third order, respectively, the solution is given by:

$$U = 1 + \eta\cos n\tilde{\theta} + \eta^2\frac{3\cos 2n\tilde{\theta}}{4(4n^2 - \bar{P}_0 - 1)}, \tag{S5}$$

$$\bar{P}_0 = (\bar{P}_0 - 3)\eta - \frac{3(12 - \bar{P}_0)}{8(15 - \bar{P}_0)}\eta^3. \tag{S6}$$

By eliminating η in these equations, we can obtain U at any angle $\tilde{\theta}$ for different deformation levels by setting a value for $\bar{P}_0$.

Ultimately, to obtain the Cartesian coordinates of the deformed tube to the undeformed angle, we obtain the following equations from the geometry of a deformed segment of the tube:

$$\frac{1}{R}\frac{dx}{d\tilde{\theta}} = -\sin\phi, \tag{S7a}$$

$$\frac{1}{R}\frac{dy}{d\tilde{\theta}} = \cos\phi, \tag{S7b}$$

where $\phi = \int_0^{\tilde{\theta}} U(\varphi)d\tilde{\theta}$. By setting x(0) = 0 and y(0) = 0 as anchoring points, these equations can be integrated to yield:

$$\frac{x(\tilde{\theta})}{R} = -\int_0^{\tilde{\theta}} \sin(\phi)d\tilde{\theta}, \tag{S8a}$$

$$\frac{y(\tilde{\theta})}{R} = \int_0^{\tilde{\theta}} \cos(\phi)d\tilde{\theta}. \tag{S9b}$$

Finally, the deformation progression of the inner tube for different numbers of WHYs n can be obtained by setting offsets from the centerline corresponding to the thickness of the inner tube t_0 (see Supplementary Fig. 8). We note that this approach, however, is incapable of solving pressure profiles that are not self-balanced. Thus, for n = 1, which causes translational movement of the inner tube, the solution can be obtained by replacing equation (S1) with the translational loading pattern in which $\bar{P} = \bar{P}_0\left(c_1 + c_2\cos\left(2\tilde{\theta}\right) + c_3\cos\left(3\tilde{\theta}\right) + c_4\cos\left(4\tilde{\theta}\right) + c_5\cos\left(5\tilde{\theta}\right) + c_6\cos\left(6\tilde{\theta}\right)\right)$. Also, while the load arising from the supply pressure could influence the geometry the inner tube, the load had negligible effect up to P_s = 120 kPa (see Supplementary Fig. 11e).

Supplementary Note 2: Maximum strain

To predict the maximum strain ε_{max} and the maximum extended length L_{max} of the STV for different numbers of WHY n, we first consider a single turn of WHY wrapped on the inner tube since the helical structure consists only of identical unit turns regardless of the number of WHY n. Next, the length of a single turn of WHY in an unwrapped form l_{1turn} can be expressed in terms of pitch p from the Pythagorean theorem[2] (see Supplementary Fig. 9a):

$$l_{1turn} = \sqrt{p^2 + (2\pi D_0)^2}, \tag{S10}$$

where D_0 is the initial distance between WHY and the initial center of the inner tube. Here, it is assumed that l_{1turn} is constant over the deformation range since the elastic modulus of WHY (~20 GPa) is much higher than that of the inner tube (~400 kPa), and the effect of the outer tube on the deformation of the inner tube is neglected. Under tensile strain $\tilde{\varepsilon}$, the pitch p increases to $p(\tilde{\varepsilon} + 1)$, and all WHYs shift closer to the initial center of the inner tube as they straighten. Thus, the distance between WHY and the initial center of the inner tube according to tensile strain $\tilde{\varepsilon}$ can be calculated from equation (S10) as follows:

$$D(\tilde{\varepsilon}) = \sqrt{\frac{{l_{1turn}}^2 - p^2(\tilde{\varepsilon}+1)^2}{4\pi^2}},$$

$$= \sqrt{{D_0}^2 - \frac{p^2\tilde{\varepsilon}(\tilde{\varepsilon}+2)}{4\pi^2}}. \quad \text{(S11)}$$

We now consider boundary conditions for different numbers of WHY n. Assuming that the thickness of the inner tube t_0 and the radius of the WHY r_0 do not change intrinsically with tensile strain, we apply geometrical boundary conditions in which the cross-sectional area of the inner tube cavity is minimized while the inner tube maintains its intrinsic thickness t_0. For example, when n = 2, the walls of the inner tube touch each other, reducing the distance between WHY to the initial center of the inner tube equal to the summation of the thickness of the inner tube t_0 and the radius of WHY r_0 at maximum strain $\tilde{\varepsilon}_{max}$. Applying tensile strain further than this point would squeeze and reduce the thickness of the inner tube extrinsically to uptake the strain. Similarly, boundary conditions for each number of WHY n are shown in Supplementary Fig. 9b and derived as follows:

$n = 1$: $$D(\tilde{\varepsilon}_{max}) = 0, \quad \text{(S12a)}$$

$n = 2$: $$D(\tilde{\varepsilon}_{max}) = r_0 + t_0, \quad \text{(S12b)}$$

$n = 3$: $$D(\tilde{\varepsilon}_{max}) = \frac{2\sqrt{3}}{3}(r_0 + t_0), \quad \text{(S12c)}$$

$n = 4$: $$D(\tilde{\varepsilon}_t) = \sqrt{2}(r_0 + t_0). \quad \text{(S12d)}$$

We note that for n = 4, this model captures the transition point $\tilde{\varepsilon}_t$ instead, where abrupt buckling of the inner tube occurs at tensile strain higher than this transition point. Then, the tensile strains $\tilde{\varepsilon}_{max}$ or $\tilde{\varepsilon}_t$ can be calculated by substituting equations (S12) into equation (S11) as follows:

$n = 1$: $$\tilde{\varepsilon}_{max} = \sqrt{\left(\frac{2\pi D_0}{p}\right)^2 + 1} - 1, \quad \text{(S13a)}$$

$n = 2$: $$\tilde{\varepsilon}_{max} = \sqrt{\left(\frac{2\pi D_0}{p}\right)^2 - 2(r_0 + t_0) + 1} - 1, \quad \text{(S13b)}$$

$n = 3$: $$\tilde{\varepsilon}_{max} = \sqrt{\frac{4\pi^2}{3p^2}(3{D_0}^2 - 4(r_0 + t_0)^2) + 1} - 1, \quad \text{(S13c)}$$

$n = 4$: $$\tilde{\varepsilon}_b = \sqrt{\frac{4\pi^2}{p^2}({D_0}^2 - 2(r_0 + t_0)^2) + 1} - 1. \quad \text{(S13d)}$$

These equations hold when strain is uniformly applied across the axial length of the inner tube,

yet stretching of the inner tube is limited at the ends by the connectors. Thus, we consider the average maximum strain ε_{max} applied to the inner tube by introducing an axial transition length L_t at both ends of the inner tube ($L_t \leq L_0/2$). This allows us to capture the drop in maximum strain ε_{max} at a lower range of L_0, where L_0 is comparable to L_t, so that the fixed ends of the inner tube exert a higher influence on restricting the stretching of the STV (see Supplementary Fig. 4g). Assuming zero strain at both ends and uniform strain $\tilde{\varepsilon}$ at the middle of the inner tube, the axial length of the inner tube L is derived from linear approximation:

$$L = 2L_t(\frac{\tilde{\varepsilon}}{2} + 1) + (L_0 - 2L_t)(\tilde{\varepsilon} + 1),$$

$$= (L_0 - L_t)\tilde{\varepsilon} + L_0. \quad \text{(S14)}$$

Finally, by substituting $L = L_0(\varepsilon+1)$ into equation (S14), we obtain the following:

$$\varepsilon = \frac{L_0 - L_t}{L_0}\tilde{\varepsilon}, \quad \text{(S15)}$$

$$\Delta L = L - L_0 = (L_0 - L_t)\tilde{\varepsilon}, \quad \text{(S16)}$$

with L_t = 15 mm fitted from the experimental data. From these equations, one can find the maximum average tensile strain ε_{max} or maximum average transition strain for buckling ε_b at any number of WHY n and pitch p by inserting equation (S13) into equation (S15), as shown in equation (2) for n = 2, for example. Furthermore, from these equations, the maximum extension length of the STV ΔL_{max} can be found at any initial length L_0 using the following equation:

$$\Delta L_{max} = L_0 \varepsilon_{max.} \quad \text{(S17)}$$

Supplementary Note 3: Considerable factors of the STV during practical use

3.1 STV design and setup

The geometrical design parameters of the STV and the setup conditions can be selected as shown in (Supplementary Fig. 5) for the use of STV in various applications.

Before the fabrication of the STV, the number of WHY n can be selected to target specific P_{ch}/P_s vs $\varepsilon/\varepsilon_{max}$ curve shape. Then, after selecting the initial length of the STV (L_0), the maximum strain ε_{max} can be determined by selecting pitch p which equivalently determines the maximum extension length of the STV (see Supplementary Note 2).

After the fabrication of the STV, the supply pressure P_s can be selected to target the maximum P_{ch}. Finally, the original/inverse STV connection mode can provide increasing/decreasing P_{ch}/P_s vs $\varepsilon/\varepsilon_{max}$ curve for the analog control of soft pneumatic actuators. The P_s and connection mode can be reprogrammed after the fabrication of the STV.

3.2 Strain rate dependence

The output chamber pressure is a strain rate-dependent function due to the intrinsic limitation in the speed of air and the inflatable characteristics of the soft pneumatic actuators. As shown in Supplementary Fig. 6a, extending the STV at faster speeds could result in reduced output chamber pressures if the air from the inlet does not have enough time to move into the actuators to achieve the target chamber pressure state. Likewise, contracting the STV at higher speeds could result in increased output chamber pressures if the air from the actuator does not have enough time to exhaust. Also, the changes in air volume required for a soft actuator to reach a specific pressure is majorly dependent on the actuator's initial chamber volume, geometry, and material properties. Since larger changes in air volume require longer time to reach the target pressure, the strain-rate dependency of the STV can be reduced, for example, by using an actuator with smaller initial chamber volume (Supplementary Fig. 6a (ii), (iii)).

In our system, the time required for the pressure to saturate for strain rate of 0.0109 s^{-1} and 0.0150 s^{-1} at $\varepsilon = \varepsilon_{max}$ was 0.11 s and 0.28 s respectively for initial chamber volume = 27,615 mm^3 and 0.3 s and 1 s respectively for initial chamber volume = 39,919 mm^3.

3.3 Longevity of the portable demo

The STV incurs a continuous loss of pressurized gas at intermediate output pressures (Supplementary Fig. 6b). We measured the flow rate of gas exhaust and found a maximum flow rate of 1.81 LPM and an average flow rate of 0.81 LPM over the strain range. We calculated the gas volume of the CO_2 canister at standard temperature and atmospheric pressure V = 52 L, with the density value obtained from the MSDS. Therefore, the portable demo last roughly 29 minutes if the STV is maintained at the highest gas loss and 64 minutes on average. The longevity could be increased by using multiple or higher capacity CO_2 canisters or/and by reducing the exhaust flow rate of the STV. We connected an inlet resistor (inner diameter of 0.4 mm and length of 20 mm) to the STV and reduced the maximum and average flow rates to 1.13 LPM and 0.51 LPM respectively. This result in increased longevity of 46 minutes at the maximum gas loss and 100 minutes on average.

3.4 Reproducibility

While an unautomated fabrication process inevitably creates variability in function, we found the simple and agile manufacturing process of the STV created negligible deviation in output data when tested with 5 different samples (Supplementary Fig. 6c).

3.5 Maximum supply pressure and the number of stretch cycles before failure

The STV is functionally active up to P_s = 140 kPa (Supplementary Fig. 4h). However, the outer tube, which has a relatively larger inner diameter, and smaller wall thickness compared to the inner tube, exponentially inflates at certain threshold supply pressure P_s (Supplementary Fig. 11e), which caused the outer tube to burst at P_s = 157 kPa. This inflation of the outer tube could be potentially blocked by adding a strain-limiting layer or tendons that exert repulsive forces on the radial expansion of the outer tube.

For the durability test, we repeatedly stretched the STV with the speed of 2 seconds per cycle and collected P_{ch}/P_s vs $\varepsilon/\varepsilon_{max}$ data at various cycles (Supplementary Fig. 6d). The STV had reliable performance up to 10,000 cycles. However, the STV faced functional degradation at

20,000 cycles and then the outer tube was detached from the connector around 25,000 cycles.

3.6 Repair after damage

Sharp cuts in the outer tube and detachment of the outer tube from the connector are two vulnerable damages that STVs may encounter during use. The STV can be functionally repaired using a non-adhesive silicone tape (HanyangMSL) for the sharp cuts of the outer tube (Supplementary Fig. 6e(i)), and silicone adhesive Silpoxy (Smooth-On) for the detachment of the outer tube from the connector tube (Supplementary Fig. 6e(ii)).

3.7 STV under different mechanical loads

The effect of bending, buckling, kinking, and compression of the STV is shown in Supplementary Fig. 7. The actuator pressure did not increase during bending, buckling, and kinking of the STV as the outer tube was only induced to wrinkle partially and the cross-section of the inner tube outer was negligibly deformed. In detail, the inlet channel resistance, which is initially very high, was not reduced to allow the inflow of air.

For the compression, the actuator pressure did not increase when STV was not strained. In detail, although the outlet resistance was significantly increased by the compression, the inlet channel resistance, which is initially very high, was not reduced to allow the inflow of air.

However, the actuator pressure increased more than the target pressure when the STV was strained and compressed by 2 mm. When the STV is strained, the WHYs deform the inner tube which reduce the inlet channel resistance and increase the outlet channel resistance. In this state, the compression significantly increased the pneumatic resistance of the outlet channel, yet the inlet channel resistance, which is not initially extremely high, was not significantly increased and allowed some flow space at the sides.

Supplementary Figures

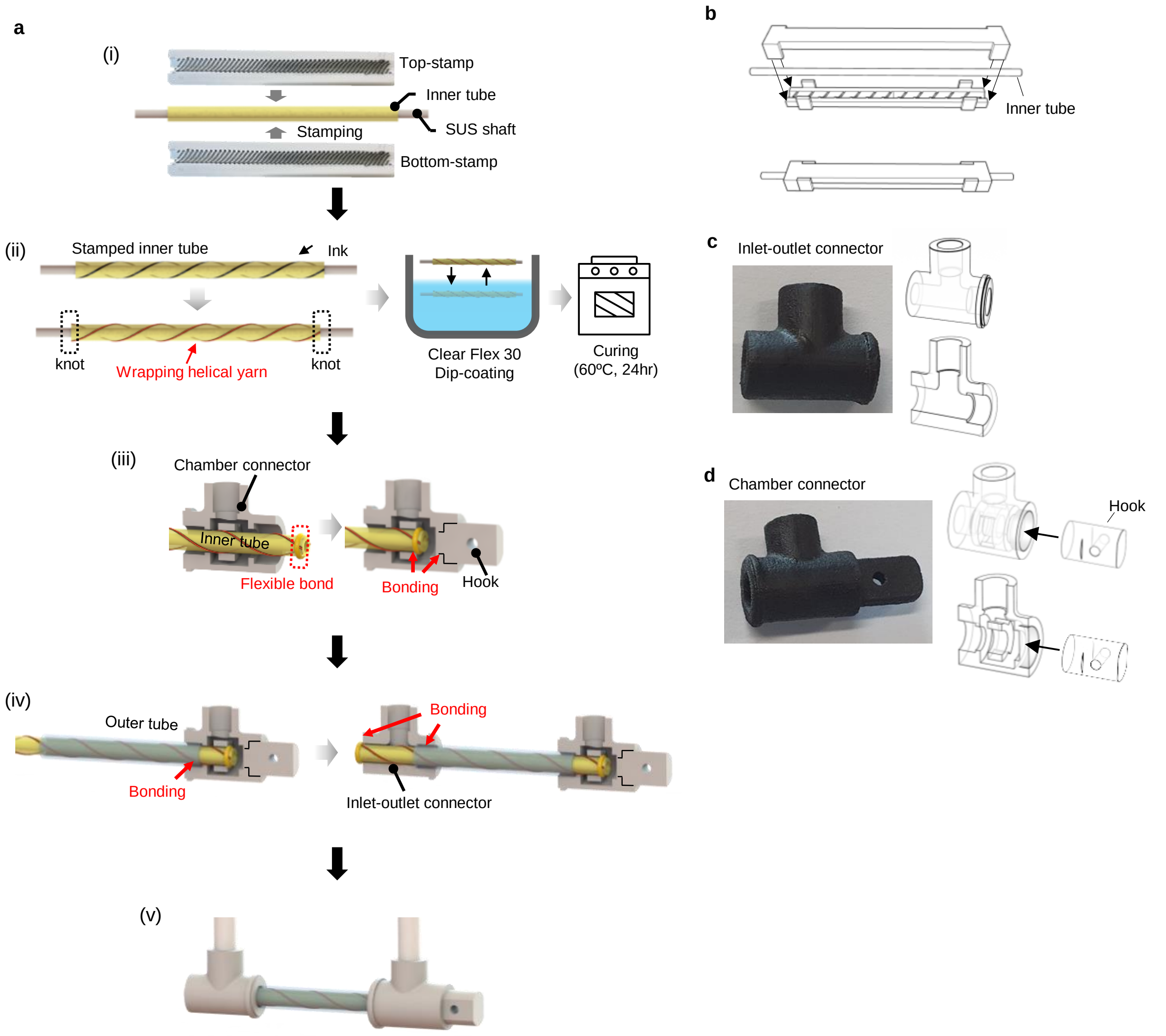

Supplementary Fig. 1. STV fabrication methods. a Schematic illustration of the overall fabrication: (i) stamping the inner tube with ink, (ii) fixing the wrapping helical yarn to the inner tube, (iii) connecting the chamber connector, (iv) fixing the outer tube and the inlet-outlet connector, and (v) fabricated STV. **b** Detailed assembly of the ink stamps for stamping. **c** and **d** Photographs of the inlet-outlet connector and chamber connector with their schematics: translucent (right-top) and projected (right-bottom).

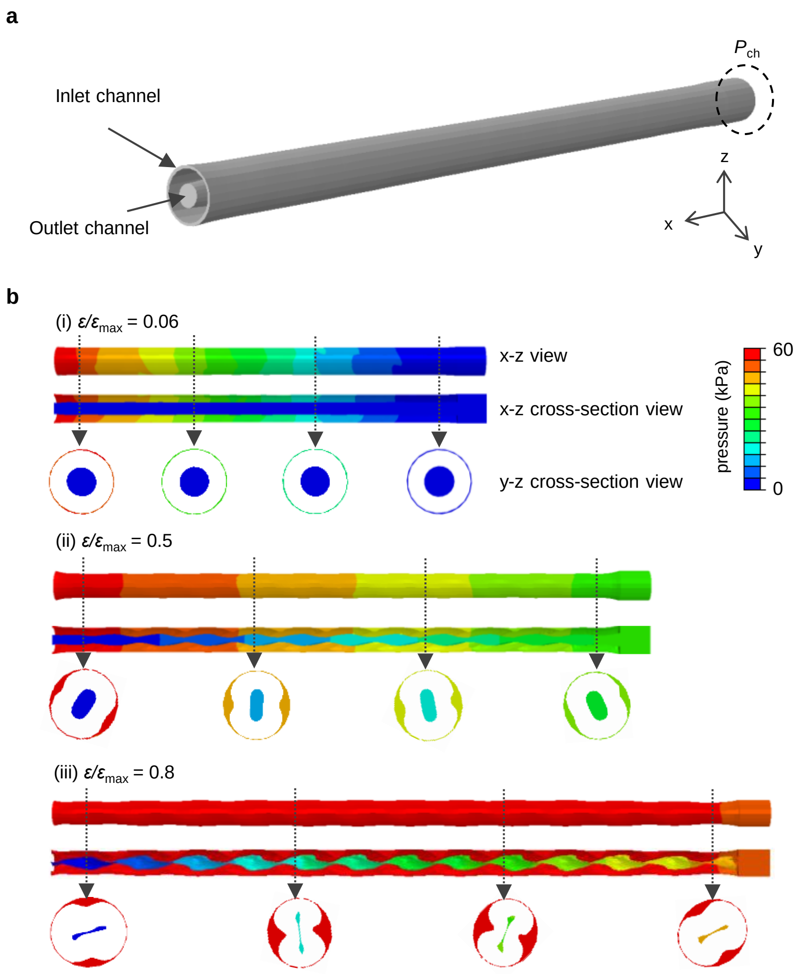

Supplementary Fig. 2. Computational fluid dynamics simulations. a Schematic illustration of the inlet and outlet channels of the STV. The dotted circle represents where the chamber pressure P_{ch} lies inside the structure. **b** Pressure profile results inside inlet and outlet channels with x-z view (top), x-z cross-sectional view (middle), and y-z cross-sectional view (bottom) at different normalized strains (i) $\varepsilon/\varepsilon_{max}$ = 0.06, (ii) $\varepsilon/\varepsilon_{max}$ = 0.5, and (iii) $\varepsilon/\varepsilon_{max}$ = 0.8.

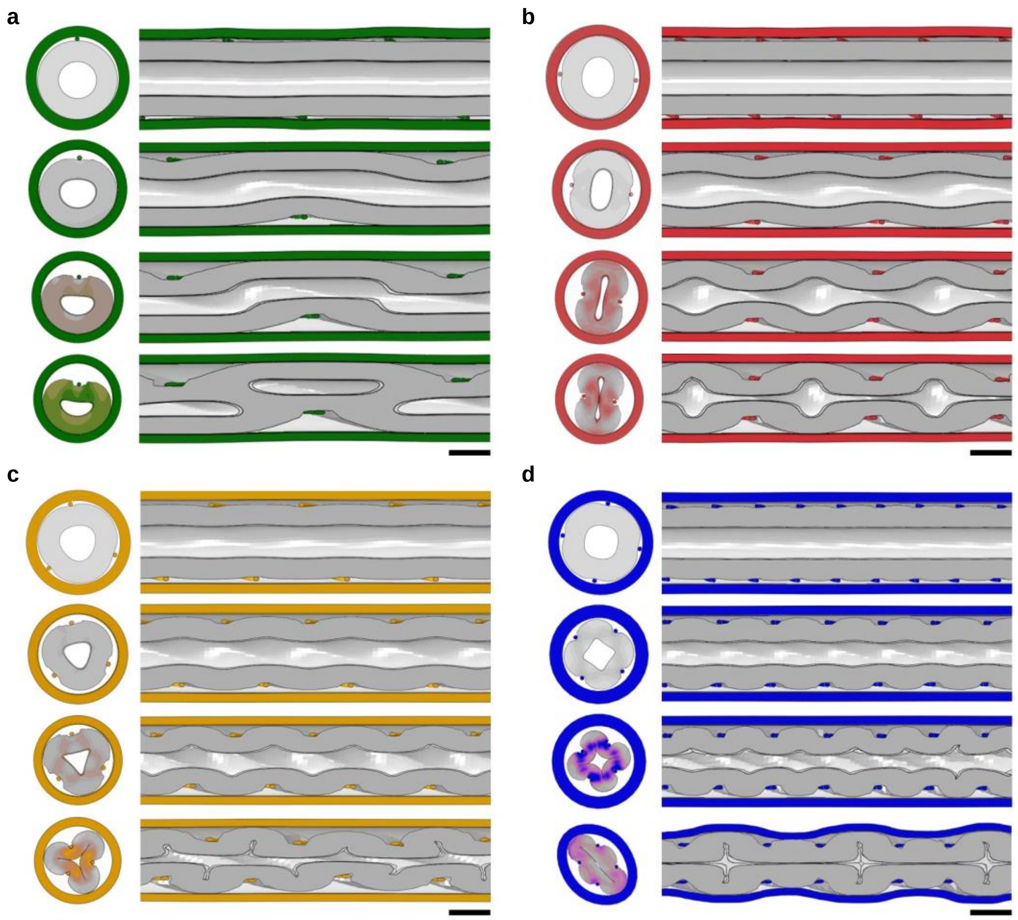

Supplementary Fig. 3. FEA results with transversal cross-sectional views at the increasing number of n. a n = 1, **b** n = 2, **c** n = 3, and **d** n = 4. Scale bars = 2 mm.

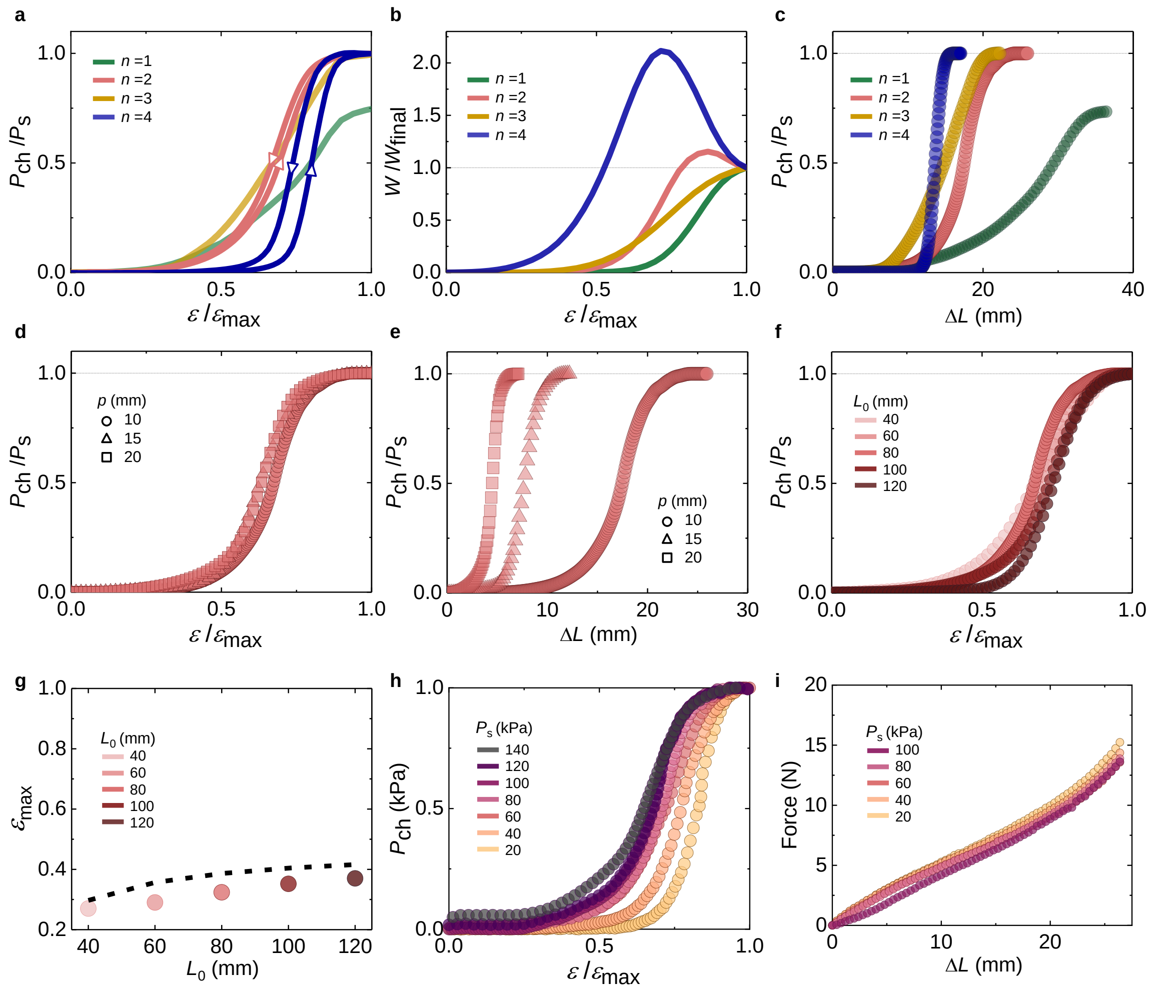

Supplementary Fig. 4. Supplementary analysis results of the STV. a Cyclic loading and unloading results of the STV shown in Fig. 2c. **b** Finite element simulation results of the normalized elastic strain energy density W/W_{max} of the inner tube as a function of the normalized strain $\varepsilon/\varepsilon_{max}$. **c** Normalized chamber pressure P_{ch}/P_s plotted against the extension length ΔL for different numbers of WHYs n; n =1 (ε_{max} = 0.46, S = 3.34), n = 2 (ε_{max} = 0.32, S = 17.26), n = 3 (ε_{max} = 0.27, S = 7.71), and n = 4 (ε_{max} = 0.21 , S = 40.66). **d** Similar chamber pressure curve profiles are obtained regardless of the cyclic pitch p. **e** Normalized chamber pressure P_{ch}/P_s plotted against the extension length ΔL for different cyclic pitch p values; p = 10 mm (ε_{max} = 0.32), p = 15 mm (ε_{max} = 0.15), and p = 20 mm (ε_{max} = 0.09). **f** Normalized chamber pressure P_{ch}/P_s as a function of normalized strain $\varepsilon/\varepsilon_{max}$ for different initial length L_0. **g** The maximum strain ε_{max} increases and then saturates as L_0 increases. The dotted line represents the analytical result (see Supplementary Note 2). The numerical values of ε_{max} for different L_0 are as follows: L_0 = 40 mm (ε_{max} = 0.27), L_0 = 60 mm (ε_{max} = 0.29), L_0 = 80 mm (ε_{max} = 0.32), L_0 = 100 mm (ε_{max} = 0.35), and L_0 = 120 mm (ε_{max} = 0.37). **h** Normalized chamber pressure P_{ch}/P_s as a function of normalized strain $\varepsilon/\varepsilon_{max}$ for different Supply pressure P_s (n = 2, p = 10 mm, L_0 = 100 mm). **i** Force plotted against the extension length ΔL for different supply pressures P_s (n = 2, p = 10 mm, L_0 = 80 mm).

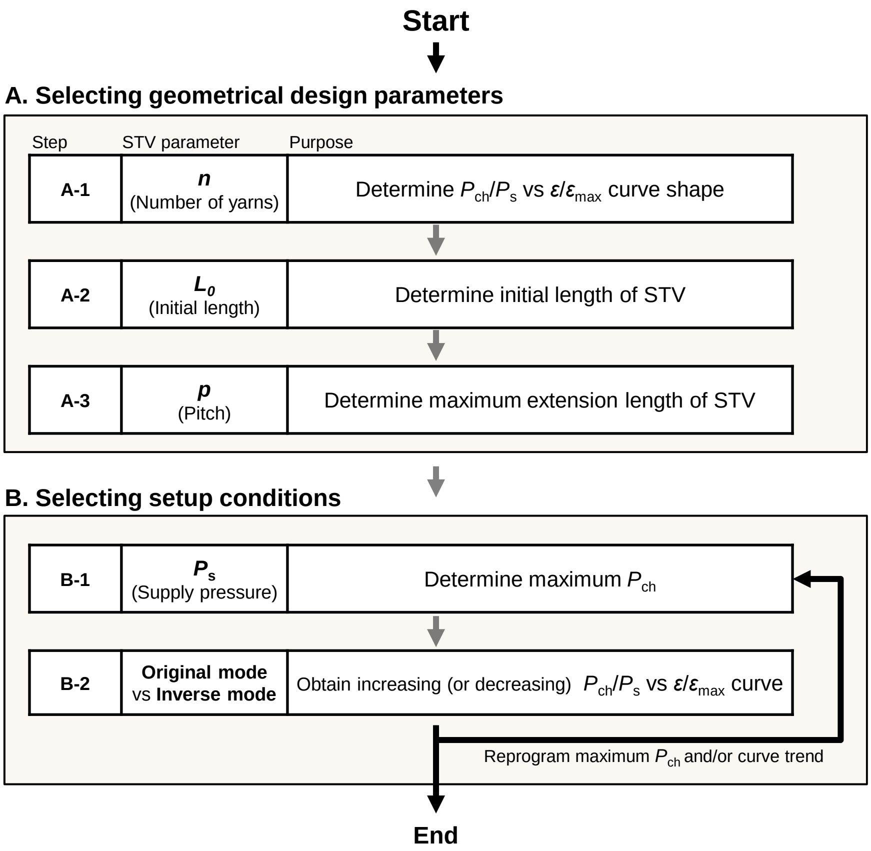

Supplementary Fig. 5. Flowchart for selecting STV geometrical parameters and setup conditions.

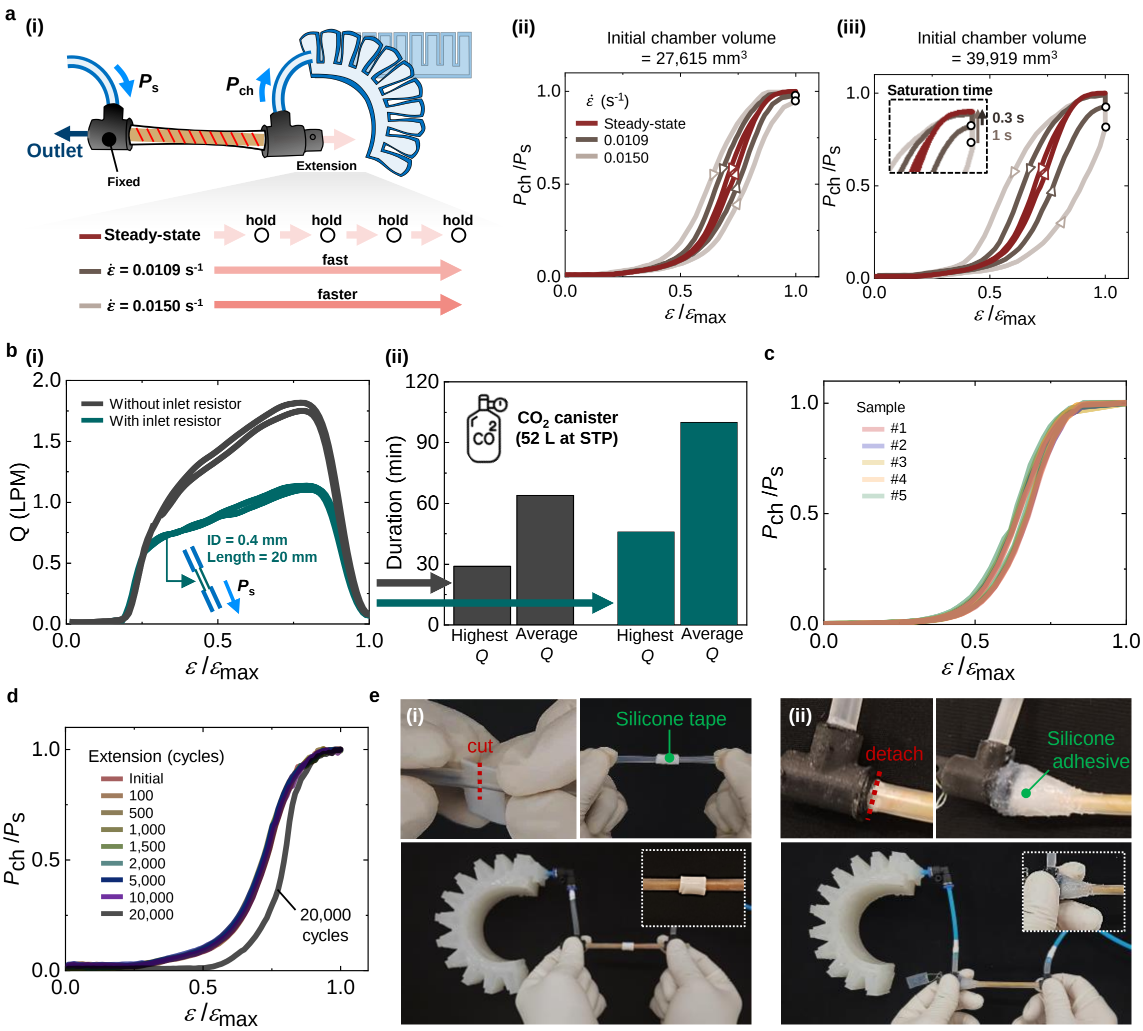

Supplementary Fig. 6. Considerable factors of the STV during practical use. a Strain rate $\dot{\varepsilon}$ dependence of the STV. (i) schematic illustration of the STV test set-up (ii) P_{ch}/P_s plotted against the normalized strain $\varepsilon/\varepsilon_{max}$ for different strain rates $\dot{\varepsilon}$ with initial actuator chamber volume of 27,615 mm^3 and (iii) 39,919 mm^3. When $\varepsilon/\varepsilon_{max} = 1$ is reached, the extension of the STV was maintained to allow the pressure inside the actuator to saturate to the target pressure. Inset: enlarged view near $\varepsilon/\varepsilon_{max} = 1$. The saturation time was (ii) 0.11 s, (iii) 0.3 s for $\dot{\varepsilon}$ = 0.0109 and (ii) 0.28 s, (iii) 1 s for $\dot{\varepsilon}$ = 0.0150 ($n = 2$, $p = 10$ mm, $L_0 = 100$ mm). **b** (i) Flow rate Q as a function of the normalized strain $\varepsilon/\varepsilon_{max}$ (Q_{max} = 1.81 LPM, $Q_{average}$ = 0.81 LPM). The inset represents inlet resistor with inner diameter of 0.4 mm and length of 20 mm connected to the inlet of the STV, reducing the flow rate to Q_{max} = 1.13 LPM, $Q_{average}$ = 0.51 LPM ($n = 2$, $p = 10$ mm, $L_0 = 100$ mm). (ii) Duration of the portable demonstrations calculated with the highest and average flow rate Q. **c** P_{ch}/P_s plotted against the normalized strain $\varepsilon/\varepsilon_{max}$ with five different samples ($n = 2$, $p = 10$ mm, $L_0 = 120$ mm). **d** P_{ch}/P_s plotted against the normalized strain $\varepsilon/\varepsilon_{max}$ after repeated extension of the STV ($n = 2$, $p = 10$ mm, $L_0 = 100$ mm, $P_s = 60$ kPa). **e** Functional repairment of the STV after failure. (i) Wrapping of non-adhesive silicone tape to repair a sharp cut of the outer tube and (ii) coating of silicone adhesive Silpoxy to repair a detachment of the outer tube from the connector.

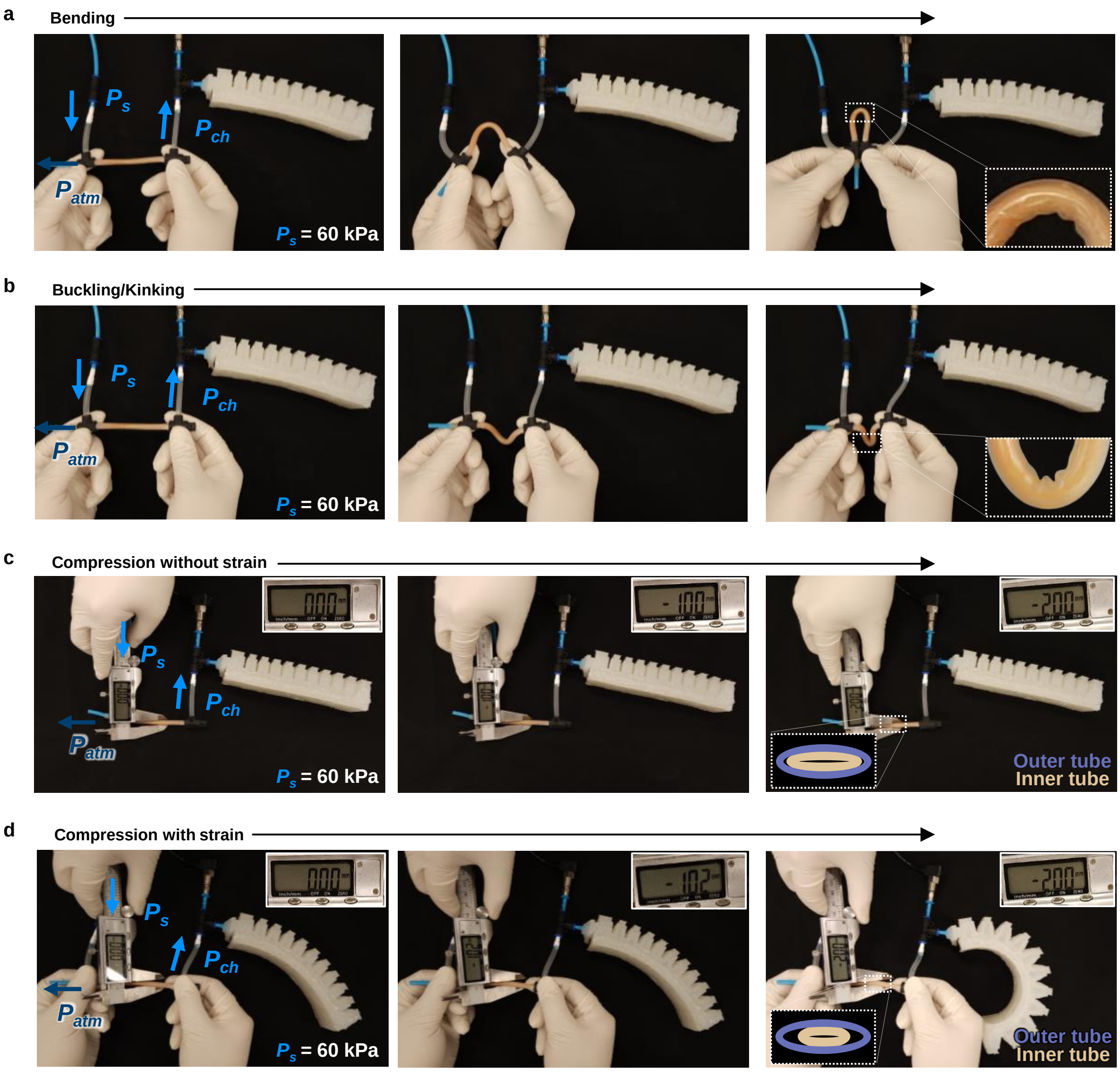

Supplementary Fig. 7. STV under different mechanical loads. Images of the STV under **a** Bending, **b** buckling and kinking (insets: enlarged images of the STV), **c** compression without extension, and **d** compression with strain (insets: schematic illustrations of the STV cross-section). The actuator pressure increased more than the target pressure only when the STV was strained and compressed 2 mm from the offset.

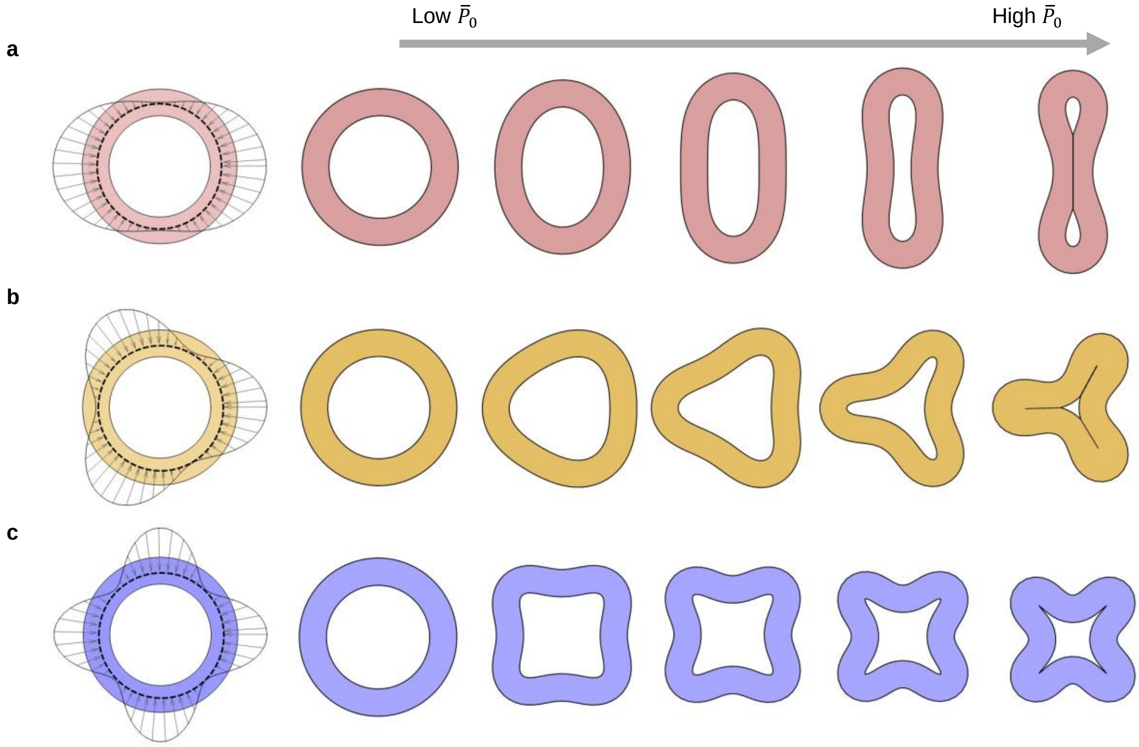

Supplementary Fig. 8. Large deformation beam theory predicting inner tube deformations. a Nonuniform pressure profile applied to the inner tube (left) and subsequent deformations results with increasing average pressure over the inner tube centerline circumference $\bar{P}_0$ (right) when the number of WHY n = 2, **b** when n = 3, and **c** when n = 4. The translational motion when n = 1 (not shown here) can be obtained using a translational loading pattern (see Supplementary Note 1).

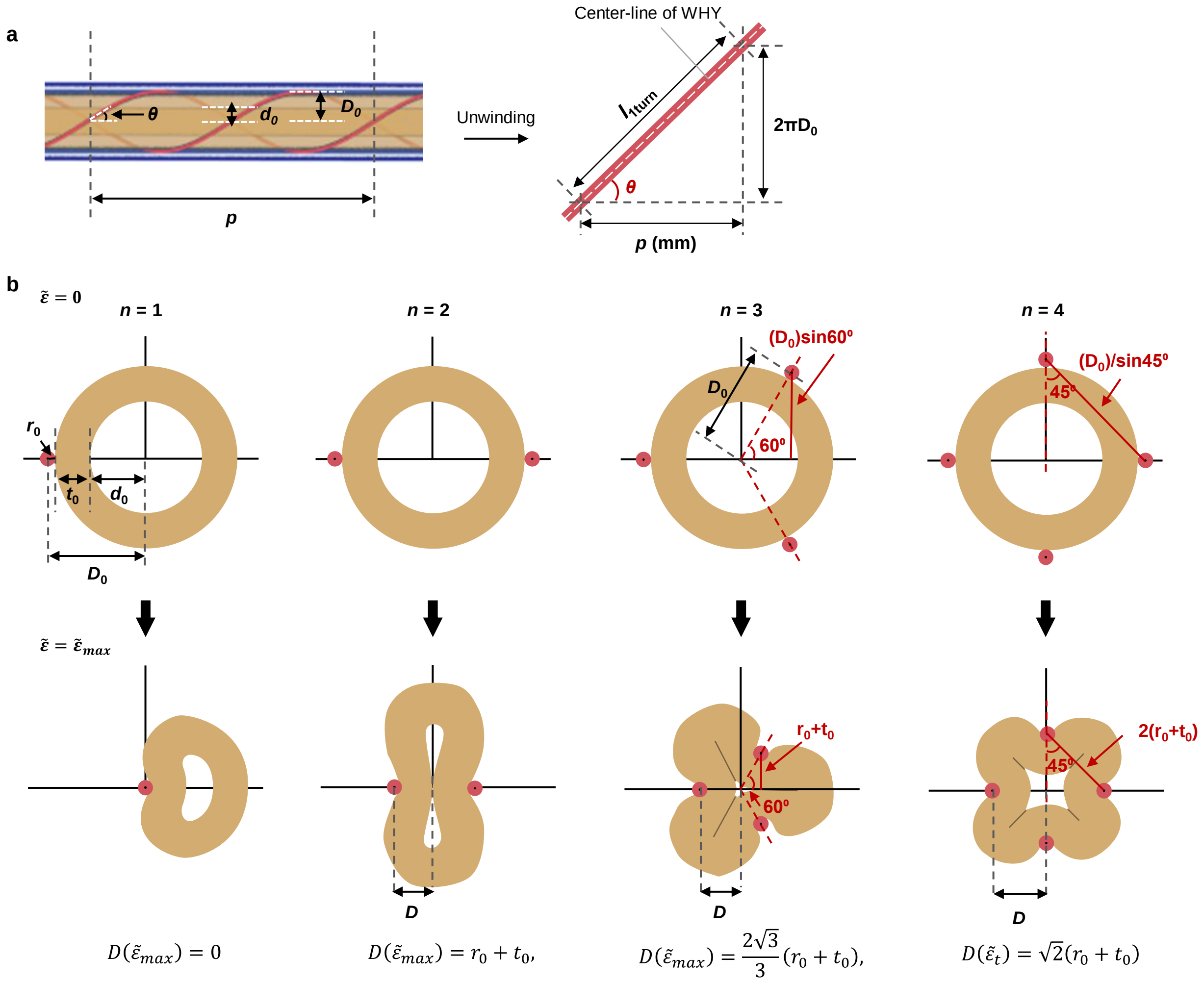

Supplementary Fig. 9. Predicting the maximum strain using Pythagorean theorem. a Schematic illustration showing the side view of the STV (left) and a single turn of the WHY in unwound form (right). **b** A schematic showing a cross-sectional view of the inner tube at tensile strain $\tilde{\varepsilon} = 0$ (top) and $\tilde{\varepsilon} = \tilde{\varepsilon}_{max}$ (bottom) for different numbers of WHYs, where $\tilde{\varepsilon}$ represents strain without accounting for fixed boundary conditions at both ends of the STV (see Supplementary Note 2).

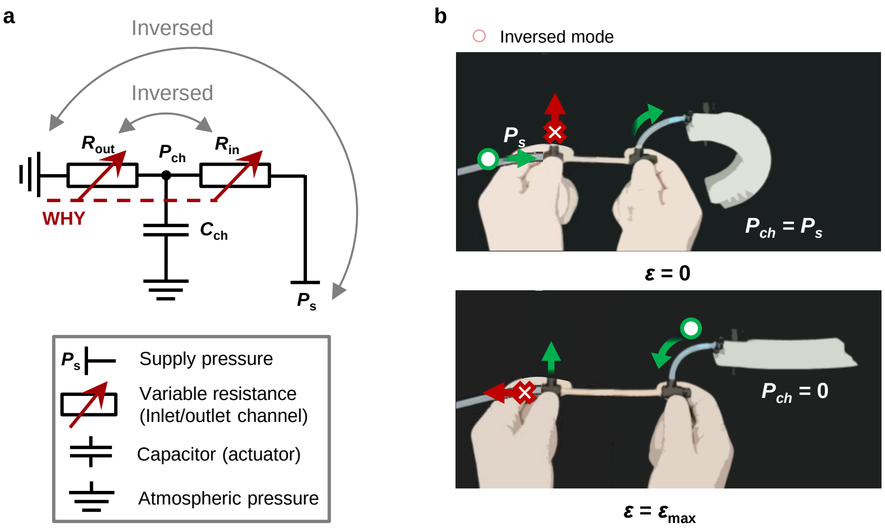

Supplementary Fig. 10. Inverse connection of the STV. a An analogous electrical circuit that represents the STV with an inverse connection. Arrows indicate inversed analogous components from the original electrical circuit in Fig. 1c. **b** Schematic demonstrations of a soft actuator controlled by the STV with an inverse connection.

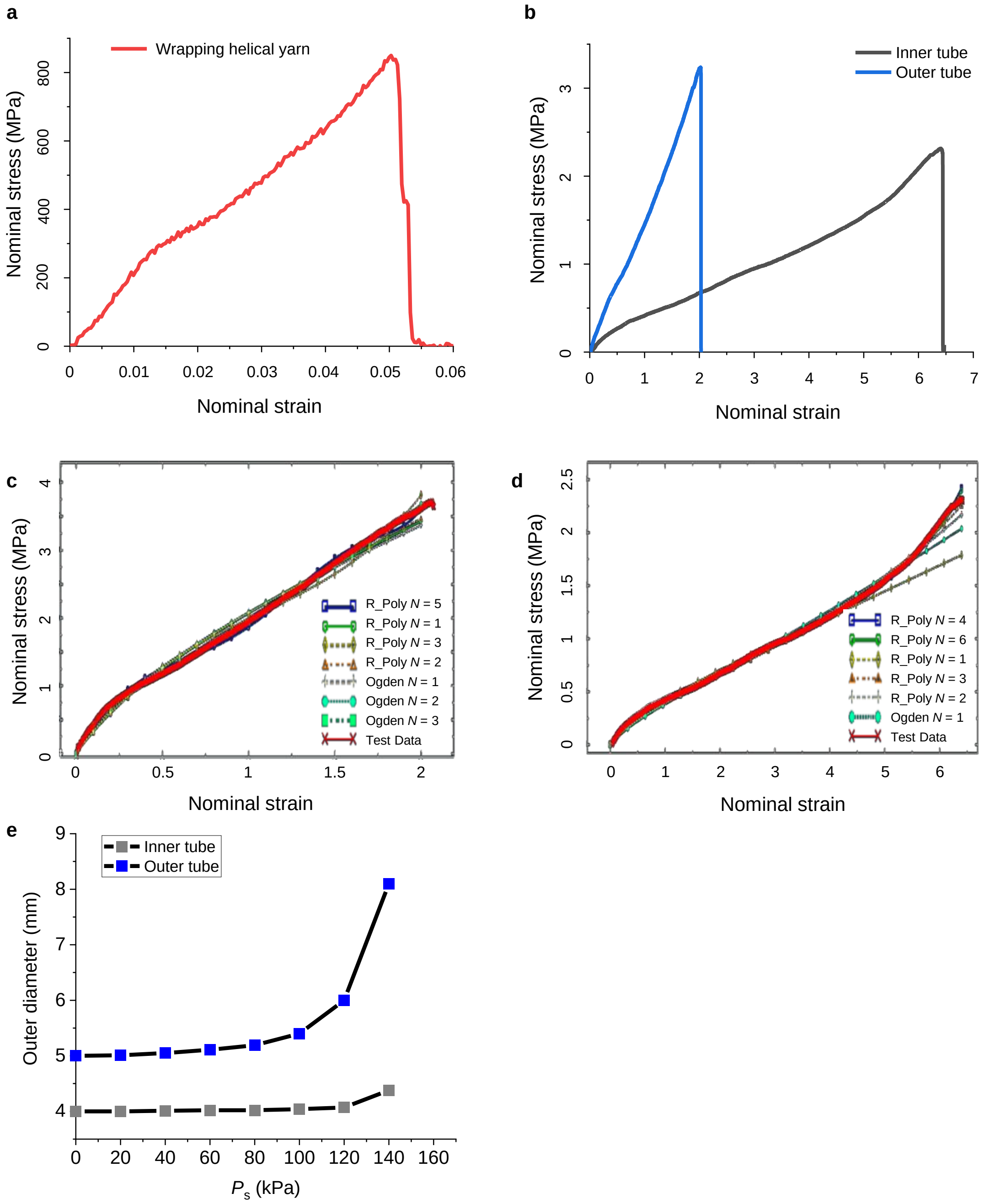

Supplementary Fig. 11. Mechanical characterizations. a Dog-bone uniaxial tensile test data of the WHY and **b** inner and outer tubes. **c** Experimental data (red bold line) and fits of various constitutive models for the outer tube and **d** for the inner tube. **e** Outer diameter of the inner and outer tube according to the supply pressure P_s.

a

b

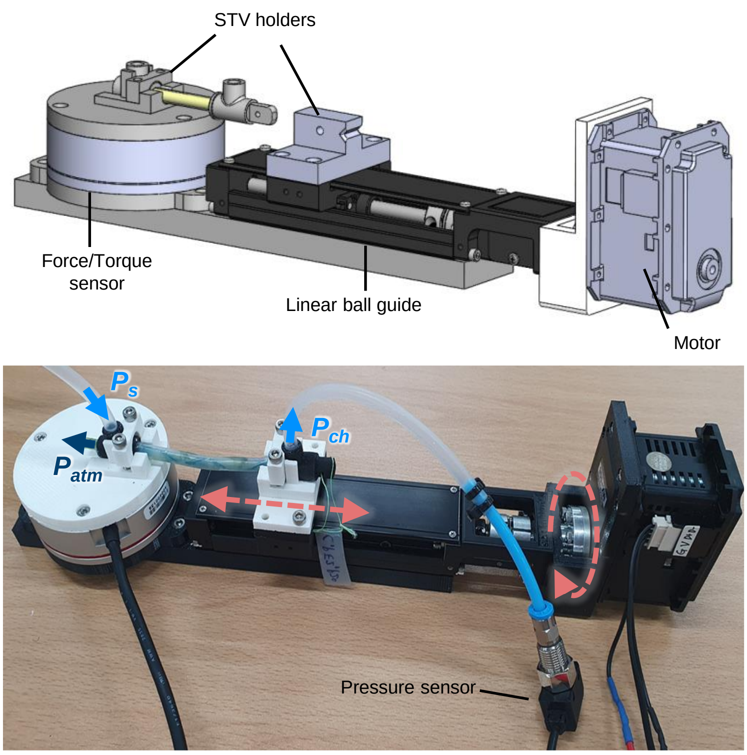

Supplementary Fig. 12. Chamber pressure curve profile analysis setup. a A schematic and **b** a photograph of the characterization components and assembly. The red dotted arrows represent linear motion of the linear ball guide (left) created by rotational motion of the motor (right).

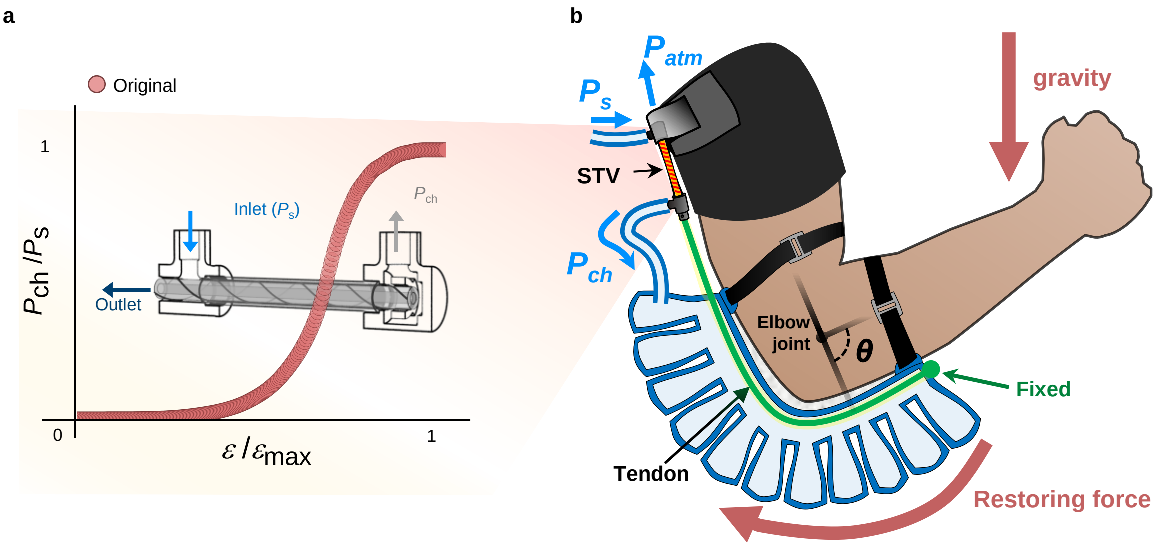

Supplementary Fig. 13. An alternative soft exosuit design with the original connection mode. a Schematic and chamber pressure curve profile of the STV showing the original connection mode. **b** A soft exosuit design with the original connection mode creating the maximum restoring force at the maximum elbow angle, which may limit the assistive torque at high elbow angles.

Capability	Our valves	Micro-Fluidics [3, 4]	Bistable silicone valves[5-8]	Digital, analog combi-native valves[9]	Bidirectional check valves[10]	Sheet-based valves[11]	Hysteretic valves[12]	Stretchable strain gauge[13]
**Mechanism**	Helical pinching	Linear pinching	Kinking	Kinking	Leaflet Flapping	Kinking	Slits in soft membrane	Channel elongation
**Device shape**	Tubular	Rectangular/ Sheet	Cylindrical	Tubular	Tubular	Sheet	Tubular	Rectangular
**Characteristic scale**	Diameter = 5 mm	Thickness = 1 ~ 2.8 mm	Diameter = 27 mm, height = 34 mm	Diameter = 15 mm*	Channel length = 4 ~ 8 mm	Thickness = 2 mm**	Diameter = 5 mm	92 x 15 x 4 (mm)***
**Practical flow rates for actuation**	✓	—	✓	✓	✓	✓	✓	—
**proportional control capabilities**	✓	—	—	✓	—	—	—	—
**Programmable proportional control**	✓	—	—	▲	—	—	—	—
**Operation with single, constant pressure source**	✓	—	✓	✓	—	✓	✓	✓
**Proportional self-sensing capabilities**	✓	—	—	✓	—	—	—	✓
**Inflow and outflow resistance control******	✓	—	—	—	—	—	—	—
**Entirely soft**	✓	✓	✓	—	✓	✓	✓	✓

* Estimated from Fig. 1c in [9]
** Estimated from Fig. S13 in [11]
*** Estimated from Fig. S13 in [13]
▲ May be capable by changing the tension in the elastic band

Supplementary Table 1. Comparison of our valves to recently developed soft control devices. **** The STV provide effective method to simultaneously modulate inflow and outflow resistance. Using a fixed outlet resistance R_{out} to achieve analog control faces a trade-off between flow leakage and switching time[52]. If R_{out} is too small, it can lead to a high loss of air and cause pump systems to fail to reach the desired pressure state. On the other hand, if R_{out} is too large, the transition of output pressures from high to low can be slowed down. Therefore, R_{out} had to be carefully set within an acceptable range, which also causes a loss of compressed gas at all output states. The STV with a helical pinching control both R_{in} and R_{out} in a programmable manner (when R_{in} increases, R_{out} decreases simultaneously and vice versa). This results in fast transitions of output from high to low with no flow leakage at high and low outputs.

	This work	Ref. [2]	Ref. [14]	Ref. [15]	Ref. [16]	Ref. [17]	Ref. [18]	Ref. [19]
**Materials**	Polyethylene yarn/ rubber tube	Spandex fiber/ silicone elastomer	Nylon/ copper wire	Kevlar fiber/ silicone elastomer	Tendon/ dexterous manipulator	Polyamide fiber/ polyurethane elastomer	Cellulose accetate,PLGA, polyurethane Nanofiber	Gold nanowire-impregnated fiber/ fiber conductor
**Type**	Pneumatic valve	Sensor	Artificial muscle	Pneumatic actuator	Continuum manipulator	Auxetics	Micro tissue	Supercapacitor
**Mechanism**	Inflow & outflow pneumatic resistance tuning	Capacitive	Radial-axial thermal expansion anisotropy	Strain limitation	Strain limitation	Interactive normal forces	Coil opening	Coil opening
**Function**	Proportional self-sensing and control of pneumatic actuators	Strain sensing	High work capacity contraction	Twist motion	S-shape motion	Negative Poisson's ratio	High stretchability	High stretchability

Supplementary Table 2. Comparison of our work to other devices using helical routing structures. Helical routing structures can be characterized by the helical arrangement of fibers or wires around a central axis. Helical tendon routing has been explored in the role of action such as capacitive strain sensing, strain limited motions, auxetic and highly stretchable shape-morphing. This work exploits helical routing for the first time in soft valves to create a compact valve with proportional self-sensing and control capabilities.

Material	Supplier	Price	Amount	Unit	Producible valves	Cost/valve
Inner tube	GSJ	$45.59	1000	g	870	0.052
Outer tube	Inner MED	$4.53	5	m	82	0.055
Polyethylene Yarn	Mark	$6.30	100	m	385	0.016
Tango rubber	Stratasys	$1518.00	3600	g	6741	0.225
Agilus rubber	Stratasys	$1628.00	3600	g	10112	0.161
Permabond 2050	Permabond	$22.20	28.3	g	353	0.062
ClearFlex30	Smooth-On	$82.00	880	g	5176	0.015
					Total cost/valve:	**$0.586**

Supplementary Table 3. Material price estimate (in USD) for the STV fabrication (n = 2, p = 10, L_0 = 80 mm).

	Viscosity (cps)	Shore A hardness	Elongation at break (%)	Coating thickness (μm)
**ClearFlex 50™**	250	50	500	10
**ClearFlex 95™**	250	95	175	10
**ClearFlex 30™**	750	30	675	50
**PMC 50™**	1400	50	500	300
**Ure-Bond ii™**	5400	85	121	600

Supplementary Table 4. Properties of commercial coating materials and resultant coating thickness. For ClearFlex 50 and ClearFlex 95, the coating thickness was insufficient to fix the WHYs on the inner tube. For PMC and Ure-Bond ii, the coating thickness was nonuniform and too thick to insert the outer tube for further fabrication. ClearFlex 30 resulted in optimal coating thickness and uniformity.

Supplementary References

1 Azzuni, E. & Guzey, S. Behavior of thin elastic circular rings with large deformations under nonuniform loads. *Journal of Pressure Vessel Technology* **141**, 011201 (2019).

2 Lee, J. *et al*. Stretchable and suturable fibre sensors for wireless monitoring of connective tissue strain. *Nature Electronics* **4**, 291-301 (2021).

3 Wehner, M. *et al.* An integrated design and fabrication strategy for entirely soft, autonomous robots. *Nature* **536**, 451-455 (2016). https://doi.org:10.1038/nature19100

4 Ranzani, T., Russo, S., Bartlett, N. W., Wehner, M. & Wood, R. J. Increasing the dimensionality of soft microstructures through injection-induced self-folding. *Advanced Materials* **30**, 1802739 (2018). https://doi.org:10.1002/adma.201802739

5 Rothemund, P. *et al.* A soft, bistable valve for autonomous control of soft actuators. *Science Robotics* **3**, eaar7986 (2018).

6 Preston, D. J. *et al.* A soft ring oscillator. *Science Robotics* **4**, eaaw5496 (2019).

7 Drotman, D., Jadhav, S., Sharp, D., Chan, C. & Tolley, M. T. Electronics-free pneumatic circuits for controlling soft-legged robots. *Science Robotics* **6**, eaay2627 (2021).

8 Preston, D. J. *et al.* Digital logic for soft devices. *Proc. Natl. Acad. Sci. USA* **116**, 7750-7759 (2019). https://doi.org:10.1073/pnas.1820672116

9 Decker, C. J. *et al.* Programmable soft valves for digital and analog control. *Proc. Natl. Acad. Sci. USA* **119**, e2205922119 (2022). https://doi.org:10.1073/pnas.2205922119

10 Davletshin, A., Underwood, T. C. & Song, W. A Bidirectional soft diode for artificial systems. *Advanced Functional Materials* **32** (2022). 2200658, https://doi.org:10.1002/adfm.202200658

11 Rajappan, A. *et al.* Logic-enabled textiles. *Proc. Natl. Acad. Sci. USA* **119**, e2202118119 (2022).

12 van Laake, L. C., de Vries, J., Malek Kani, S. & Overvelde, J. T. B. A fluidic relaxation oscillator for reprogrammable sequential actuation in soft robots. *Matter* **5**, 2898-2917 (2022). https://doi.org:10.1016/j.matt.2022.06.002

13 Koivikko, A. *et al.* Integrated stretchable pneumatic strain gauges for electronics-free soft robots. *Communications Engineering* **1**, 14 (2022). https://doi.org:10.1038/s44172-022-00015-6

14 Haines, C. S. *et al*. New twist on artificial muscles. *Proc. Natl. Acad. Sci. USA* **113**, 11709-11716 (2016).

15 Geer, R., Iannucci, S. & Li, S. Pneumatic coiling actuator inspired by the awns of erodium cicutarium. *Frontiers in Robotics and AI* **7**, 17 (2020).

16 Gao, A. *et al*. in 2015 *IEEE/RSJ International Conference on Intelligent Robots and Systems (IROS)*, 2012-2017 (IEEE).

17 Sloan, M. R., Wright, J. R. & Evans, K. E. The helical auxetic yarn – A novel structure for composites and textiles; geometry, manufacture and mechanical properties. *Mechanics of Materials* **43**, 476-486 (2011).

18 Li, Y. *et al*. Helical nanofiber yarn enabling highly stretchable engineered microtissue. *Proc. Natl. Acad. Sci. USA* **116**, 9245-9250 (2019).

19 Zhao, Y. *et al*. Highly stretchable fiber-shaped supercapacitors based on ultrathin gold nanowires with double-helix winding design. *ACS Appl. Mater. Interfaces* **10**, 42612-42620 (2018).
