# Supplementary material for: A soft, self-sensing tensile valve for perceptive soft robots
Source: Nat Commun. 2023 Jul 4;14:3942. doi: 10.1038/s41467-023-39691-z (PMC10319868; doi:10.1038/s41467-023-39691-z)
Supplement: Supplementary file 3 — Description of Additional Supplementary Files [file 41467_2023_39691_MOESM3_ESM.pdf]

Description of Additional Supplementary Files

Supplementary Movie 1: Analog control of a soft actuator. The STV is connected to a constant supply pressure and a soft actuator. The soft actuator bends proportionally and continuously to the strain applied to the STV.

Supplementary Movie 2: Finite element method extension simulations. A translucent view of the assembled tubes and the inner tube upon extension. Cross-sectional view of the STV upon extension for different number of WHY n. Side view of the inner tube for different cyclic pitch p and initial length traced by the centerline of WHY L0.

Supplementary Movie 3: STV inverse connection. Comparison between two different modes: (i) original STV connection mode with an increasing pressure curve upon tensile strain, and (ii) inverse STV connection mode with an inversed, decreasing pressure curve upon the tensile strain.

Supplementary Movie 4: Untethered and electronics-free soft gripper. Gripping objects with various shapes, weights, and fragility. Untethered and Electronics-free demonstration of the gripper. Gripping demonstration of an underwater object.

Supplementary Movie 5: Autonomous and self-adaptive exosuit. The exosuit autonomously adjusts the elbow assist torque by controlling the chamber pressure of the soft elbow actuator according to the elbow angle. Characterization setup using a mannequin with a motor and a torque sensor embedded in the elbow. Measured elbow torques required to lift the forearm over the useful range of motion of the elbow with different mannequin conditions: (i) undressed, (ii) soft elbow actuator with constant Pch = 80 kPa, and (iii) our exosuit with STV control.
